# Evaluation of Antigen-Conjugated Fluorescent Beads to Identify Antigen-Specific B Cells

**DOI:** 10.3389/fimmu.2018.00493

**Published:** 2018-03-23

**Authors:** Isabel Correa, Kristina M. Ilieva, Silvia Crescioli, Sara Lombardi, Mariangela Figini, Anthony Cheung, James F. Spicer, Andrew N. J. Tutt, Frank O. Nestle, Panagiotis Karagiannis, Katie E. Lacy, Sophia N. Karagiannis

**Affiliations:** ^1^St. John’s Institute of Dermatology, School of Basic & Medical Biosciences, King’s College London, Guy’s Hospital, London, United Kingdom; ^2^NIHR Biomedical Research Centre at Guy’s and St. Thomas’s Hospitals and King’s College London, King’s College London, London, United Kingdom; ^3^Breast Cancer Now Research Unit, School of Cancer & Pharmaceutical Sciences, King’s College London, Guy’s Cancer Centre, London, United Kingdom; ^4^Department of Applied Research and Technology Development, Fondazione IRCCS Istituto Nazionale dei Tumori, Milan, Italy; ^5^School of Cancer & Pharmaceutical Sciences, King’s College London, Guy’s Hospital, London, United Kingdom; ^6^Breast Cancer Now Toby Robins Research Centre, Institute of Cancer Research, London, United Kingdom; ^7^Immunology and Inflammation Therapeutic Research Area, Sanofi US, Cambridge, MA, United States; ^8^Department of Oncology, Haematology and Stem Cell Transplantation, University Hospital of Hamburg Eppendorf, Hamburg, Germany

**Keywords:** B cell, humoral immune response, single cell sorting, fluorescent bead, human antibodies, antibody expression, antigen

## Abstract

Selection of single antigen-specific B cells to identify their expressed antibodies is of considerable interest for evaluating human immune responses. Here, we present a method to identify single antibody-expressing cells using antigen-conjugated fluorescent beads. To establish this, we selected Folate Receptor alpha (FRα) as a model antigen and a mouse B cell line, expressing both the soluble and the membrane-bound forms of a human/mouse chimeric antibody (MOv18 IgG1) specific for FRα, as test antibody-expressing cells. Beads were conjugated to FRα using streptavidin/avidin-biotin bridges and used to select single cells expressing the membrane-bound form of anti-FRα. Bead-bound cells were single cell-sorted and processed for single cell RNA retrotranscription and PCR to isolate antibody heavy and light chain variable regions. Variable regions were then cloned and expressed as human IgG1/k antibodies. Like the original clone, engineered antibodies from single cells recognized native FRα. To evaluate whether antigen-coated beads could identify specific antibody-expressing cells in mixed immune cell populations, human peripheral blood mononuclear cells (PBMCs) were spiked with test antibody-expressing cells. Antigen-specific cells could comprise up to 75% of cells selected with antigen-conjugated beads when the frequency of the antigen-positive cells was 1:100 or higher. In PBMC pools, beads conjugated to recombinant antigens FRα and HER2 bound antigen-specific anti-FRα MOv18 and anti-HER2 Trastuzumab antibody-expressing cells, respectively. From melanoma patient-derived B cells selected with melanoma cell line-derived protein-coated fluorescent beads, we generated a monoclonal antibody that recognized melanoma antigen-coated beads. This approach may be further developed to facilitate analysis of B cells and their antibody profiles at the single cell level and to help unravel humoral immune repertoires.

## Relevance to the Research Topic: Single Cell Approaches to Study the Immune System

The selection and study of single antigen-specific B cells and the identification of their expressed antibodies can help shed light on the nature and functions of the human humoral immune response. In our study, we present an antigen-conjugated fluorescent bead methodology designed to identify and isolate single antibody-expressing cells and to derive and clone matched heavy and light chain antibody variable regions into full length antibodies. We believe that our single cell-to-functional antibody strategy falls within the scope of this research topic, as it opens the way for opportunities to analyze B cells and their antibody profiles at the single cell level and may be potentially applied to help unravel diverse humoral immune repertoires in different health and disease states.

## Introduction

The study of humoral immunity may be key to understand human health, aging, and disease, as well as to predict or monitor host immune responses to pathogen challenge and disease progression (1). Moreover, dissecting B cell responses could help inform medical interventions such as preventative vaccines or therapeutic treatments (2, 3). Currently, therapeutic monoclonal antibodies are part of the standard care of treatment for various conditions including diabetes, autoimmune and allergic diseases, and various types of cancer. A big proportion of these antibodies are fully mouse or mouse–human chimeras (4). Therefore, identification of fully human, heavy (H), and light (L) variable region-matched antibodies may be of interest for clinical applications, as administration of fully human antibodies is considered less likely to induce antidrug antibody responses compared with chimeric or humanized antibodies (5). Immune monitoring of human B cells recognizing specific antigens, alongside identification, cloning, and production of their cognate monoclonal antibodies, may, therefore, be of substantial value to help evaluate reactivity and immunological response to these antigens.

Analyses of the antibody repertoires of B cells have been reported in the human setting and in animal models, with the most common applications in the study of autoimmune diseases (6), viral infections, and B cell malignancies (7–9). Some studies have employed high-throughput sequencing of heavy chain variable regions or of paired H and L variable region sequences (10, 11). Ultra-high-throughput sequencing techniques have also been developed, capable of analyzing repertoires of thousands of B cells from small human blood samples, with some allowing the mapping of paired H and L variable regions, in a few hours (12–14).

B cells may also be a source of antigen-specific antibodies for clinical diagnostics or for therapeutic applications. Existing methods to identify antigen-specific B cells and generate monoclonal antibodies include *ex vivo* B cell culture approaches, which could promote the survival and expansion of certain B cell subsets, screening of the culture supernatants to identify B cell reactivity and fluorescent-activated cell sorting (15–20). An essential element in the process of selecting antigen-specific B cells is detection of antibodies with a certain degree of specificity. This could be achieved by screening cell culture supernatants through ELISPOT assays or ELISA-based methods using immobilized recombinant antigens or cells (16, 20). Screening cell culture supernatants by ELISA, although highly sensitive, represents only a surrogate parameter and antigen reactivity should ultimately be confirmed only after sequencing and expression of the selected clone. For all these applications, the gold standard of identifying antigen-specific antibodies remains the expression of the recombinant antibody and further evaluation of its antigen recognition properties. Workflows to facilitate selection of single human B cells without *ex vivo* growth, stimulation, and clone expansion, and which do not require sampling of cell culture supernatants could offer additional tools for the study of human B cell immunity. Novel approaches to address these requirements involve the use of modified fluorescent tetramers for direct B cell screening by fluorescent-activated cell sorting (21, 22).

In this study, we describe the design of a bead-based methodology to identify single antibody-expressing B cells, and to clone and produce antigen-specific antibodies. The workflow features bead-based identification and isolation of specific B cells using direct fluorescent-activated cell sorting, sequencing, and cloning of matched heavy and light chain variable regions in a single full sequence antibody expression vector system, and expression and testing the antigenic reactivity of the antibody clone. The workflow is designed to avoid *ex vivo* B cell expansion and secondary clone selection and to facilitate antibody generation and downstream evaluation.

## Materials and Methods

### Human Samples

Human immune cells were isolated from venous blood of healthy volunteers and patients with malignant melanoma. Specimens were collected with informed written consent in accordance with the Declaration of Helsinki. The study was conducted at King’s College London, King’s College London, Guy’s and St Thomas’ NHS Foundation Trust (08/H0804/139 approved by London Bridge NRES committee; 16/LO/0366 approved by London-Central NRES Committee). Human peripheral blood mononuclear cells (PBMC) were isolated from 40 ml blood using Ficoll^®^ Paque Plus density centrifugation (GE Healthcare).

### Cell Culture

Cell culture was performed using aseptic technique at 37°C in a humidified atmosphere in 5% CO_2_, unless otherwise specified. The human ovarian carcinoma cell line IGROV1 naturally over-expressing folate receptor alpha (FRα) was grown in RPMI 1640 GlutaMAX™ medium (Thermo Scientific) supplemented with 10% fetal calf serum (FCS). The human breast cancer cell line MDA-MB-231 was grown in DMEM GlutaMAX™ medium (Thermo Scientific) supplemented with 10% FCS. The permanently transfected murine myeloma cell lines SP2/0-MOv18 specific for FRα and SP2/0-SF25, recognizing a colon carcinoma antigen (23), were cultured in Dulbecco’s Modified Eagle’s Medium plus 10% FCS as previously described (24). The human embryonic kidney cell lines, Expi293F cells, were cultured in serum-free Expi293 expression medium (Thermo Scientific) on a Stuart orbital shaker at 125 rpm at 8% CO_2_.

### Transient Expression of Human Monoclonal Antibodies in Expi293F Cells

Expi293F cells were transfected with pVitro1-hygro-mcs antibody constructs using the ExpiFectamine293 Transfection kit (Thermo Scientific) as per manufacturer’s instructions. The anti-human epidermal growth factor receptor 2 (HER2) and the melanoma-associated antigen-specific chondroitin sulfate proteoglycan (CSPG4) antibody constructs were previously described (25, 26).

### Fluorescent Beads

Different avidin- or streptavidin-coated fluorescent beads of different sizes were used (Table S1 in Supplementary Material): XMAP LumAvidin Microspheres (LumAvidin 5.6 µm) (L100-L150-01) with a size of 5.6 µm and fluorescent in the APC channel (from Luminex); Sphero Coated-fluorescent particles (Spherotec Inc.) as follows: Sphero Streptavidin-coated fluorescent particles, Nile Red 0.4–0.6 µm (SA-Red 0.5 µm) (Cat No SVFP-0556-5), Sphero Avidin-coated fluorescent particles Nile Red, 0.7–0.9 µm (A-Red 0.8 µm) (Cat No VFP-0856-5), and Sphero Streptavidin-Coated fluorescent particles, Blue, 1.0–1.9 µm (SA-Blue 1.1 µm) (Cat No SVFP-1068-5).

### Generation of Melanoma Cell Line Protein Extracts

To biotinylate proteins of melanoma cell lines, confluent cell monolayers were washed with PBS and borate buffer pH 9. Then, sulfo-NHS-LC-biotin at 1 mg/ml in PBS was added and incubated for 20 min on ice. The reaction was quenched with 200 mM Tris, 120 mM NaCl pH 7.4, and the cells were subsequently extensively washed with PBS. Cell lysis buffer (Cell Signaling Technology) containing 0.1% Triton and supplemented with 2 mM PMSF was added to the monolayer and the cells were harvested by scrapping. Lysates were briefly sonicated and incubated on an orbital rocker for 30 min at 4 C. The lysates were cleared by centrifugation at 900 × *g* for 15 min, followed by a second centrifugation at 12,000 × *g* for 30 min. Supernatants were either used immediately or stored at −80°C.

### Coupling of Recombinant Proteins to Fluorescent Beads

Recombinant Folate Receptor α (FRα) (R&D Systems, Cat no 5646-FR) and human epidermal growth factor receptor 2 (ErbB2/HER2) Fc chimera (R&D Systems, Cat no 1129-ER) were reconstituted in PBS at 0.1 mg/ml as recommended by the manufacturer. Biotins with three different arms were used: (i) sulfo-NHS-LC-biotin with a spacer arm of 22.4 Å; (ii) sulfo-NHS-LC-LC-biotin with a spacer arm of 30.5 Å, and (iii) NHS-PEG12-biotin with a spacer arm of 56 Å (all from Pierce-Thermo Fisher Scientific). The three forms of biotin were reconstituted following the manufacturer’s instructions and mixed with the recombinant proteins in PBS at a 20:1 (biotin:recombinant protein) molar ratio and incubated for 90 min at room temperature (RT). Free biotin was removed by dialysis in PBS.

For binding of biotin-conjugated FRα to LumAvidin 5.6 µm microspheres, 100 µl of microsphere solution was washed with 1 ml of 1% BSA–PBS–0.05% sodium azide, spun at 8,000 × *g* for 2 min, and pellets were resuspended in 100 µl of 1% BSA–PBS–0.05% sodium azide. Then, 37.5 µg of biotin-conjugated recombinant protein (in 300 µl of PBS-1% BSA) was added to the beads and mixed, by rotation, for up to 16 h at RT. Beads were then washed three times with 1 ml of 1% BSA–PBS–0.05% sodium azide and resuspended in 100 µl of 1% BSA–PBS–0.05% sodium azide.

For binding of biotin-conjugated FRα to all Sphero fluorescent particles, 100 µl of beads were washed with 1 ml of 1% BSA–PBS–0.05% sodium azide, spun at 13,000 × *g* for 20 min at RT, and the pellet was resuspended in the original volume of beads with 1% BSA–PBS–0.05% sodium azide. Then, 25 µg of biotin-conjugated recombinant protein was added and mixed, by rotation, for 2–16 h at RT. After binding, beads were washed three times with 1 ml 1% BSA–PBS–0.05% sodium azide and resuspended in 100 µl of 1% BSA–PBS–0.05% sodium azide.

For binding of biotin-conjugated cell extracts to LumAvidin 5.6 µm microspheres, 200 µl of microsphere solution were washed with 1 ml of 1% BSA–PBS–0.05% sodium azide, spun at 8,000 × *g* for 2 min, and pellets were resuspended in 200 µl of 1% BSA–PBS–0.05% sodium azide. Then, 300 µl of biotin-conjugated cell extracts were added to the beads and mixed, by rotation, for up to 16 h at RT. After binding, beads were washed three times with 1 ml of 1% BSA–PBS–0.05% sodium azide and resuspended in 200 µl of 1% BSA–PBS–0.05% sodium azide. For each test, 15 µl beads were used.

### Fluorescent Labeling of Conjugated Beads

To check that FRα/HER2 proteins were attached on the bead surface, conjugated beads were stained with specific antibodies for FRα/HER2. Conjugated beads (10 µl) were incubated in 200 µl of FACS buffer (5% FCS–PBS) for 20 min at RT, then, 1 µg of the monoclonal antibody MOv18 (anti-FRα), anti-HER2 [previously described in Ref. (27)] or isotype control antibodies were added. In all described experiments, the hapten-specific in-house produced monoclonal antibody anti-NIP IgG was used as an isotype control. The beads were incubated for 30 min at 4°C, washed with 2 ml of FACS buffer, and incubated with anti-human immunoglobulin (Ig) antibody conjugated to FITC (Vector Laboratories) for another 30 min at 4°C. After a final wash with FACS buffer, stained beads were analyzed on a FACSCanto™ flow cytometer (BD Biosciences). Beads coated with melanoma cell line extracts were tested by staining with a monoclonal IgG antibody recognizing the melanoma-associated antigen CSPG4.

### Binding of Conjugated Beads to Cells Expressing MOv18 or Anti-HER2 Antibodies

Sp2/0 B cells transfected with MOv18 IgG1 antibody or Expi293F cells transfected to transiently express anti-HER2 antibodies (10^5^ cells in 100 µl of FACS buffer) were incubated with 2–30 µl of conjugated beads for 30–45 min at RT, washed with 2 ml of FACS buffer, and left on ice until analyzed on a FACSCanto™ flow cytometer (BD Biosciences).

### Spiking of PBMC with Antibody-Expressing Cells

Sp2/0 cells expressing MOv18 IgG or the control antibody SF25 were prestained with anti-mouse MHC Class I-PE antibody to be used with Streptavidin SA-Blue 1.1 µm or LumAvidin 5.6 µm beads (Table S1 in Supplementary Material) or anti-mouse CD45 APC antibody (BD Biosciences)/Far-red cell tracking dye (Thermo Fisher Scientific) to be used with Avidin fluorescent A-Red 0.8 µm beads. Expi293F cells expressing anti-HER2 or the control anti-CSPG4 antibody (3–4 days post transfection) were labeled with Far-red cell tracking dye (Thermo Fisher Scientific). Beads coated with recombinant antigens were incubated with 100 µl of blocking buffer (10% FCS–PBS, unless stated) for 30 min at RT. Then, 10^6^ PBMCs were added in 100 µl of blocking buffer and spiking was conducted with pre-labeled Sp2/0-or Expi293F cells at different PBMC:pre-labeled cell ratios in another 50 µl. Cells and beads were incubated for 30 min at 4°C, washed with 2 ml of FACS buffer, and stained with DAPI before samples were analyzed on a FACSCanto™ flow cytometer (BD Biosciences). The data were analyzed using the FlowJo^®^ software version 10.4.1. The actual dilution was calculated by dividing the number of live Far-red positive events (antigen-specific cells) by the number of live Far-red negative events (PBMC).

### Cell Sorting

Sp2/0 cells transfected with MOv18 IgG and stained with FRα-conjugated fluorescent beads were sorted on 96-well plates at 1, 5, and 10 cells/well directly into 10 µl of lysis buffer (Single cell lysis kit, Ambion-Thermo Fisher Scientific), using an BD FACSAria™ II Cell Sorter (BD Biosciences) with an 85 µm nozzle. Samples were transferred to 0.2 ml PCR tubes and stored at −80°C until further processing. Enriched B cells were obtained from 40 ml peripheral blood of two patients with melanoma using RosetteSep human B cell enrichment cocktail (StemCell Technologies) following the manufacturer’s instructions. B cells were incubated with Fc Blocking Reagent (Miltenyi Biotec) for 10 min at RT and stained with anti-CD19 FITC, anti-CD22 FITC, anti CD45 PE (all antibodies from BD Biosciences) and simultaneously incubated with 15 µl of 5.6 µm LumiAvidin beads coated with SK-MEL-28 melanoma cell extracts for 30 min on ice in sort buffer (PBS, 2% BSA, 2 mM EDTA). Immediately before acquisition, DAPI live/dead dye was added. The cells binding fluorescent beads were sorted into 72-well plates at 1 cell/well into 10 µl of lysis buffer as above. For B cells, a 70 µm nozzle was used.

### Ig Variable Region Cloning

PCR tubes containing sorted cells in lysis buffer were thawed and reverse transcription was performed in a thermal cycler using the SuperScript VILO cDNA Synthesis kit (Thermo Fisher Scientific) following the manufacturer’s recommendations. Variable regions of Ig heavy and light chains were amplified by two rounds of PCR amplification in 20 µl mix, using 10 µl of Phusion Flash polymerase mix per reaction (Finnzymes), each primer at 0.5 µM, 2 µl of the reverse transcriptase reaction as template for the first PCR and 2 µl of the first PCR as template in the second PCR. For the variable regions in the MOv18 heavy chain, the cycles for amplification were: 1 min at 98°C, 36 cycles of 10 s at 98°C, 15 s at 60°C, and 15 s at 72°C, followed by a final step at 72°C for 1 min. In the first PCR, the reverse primer was IgGRev in the constant region of the IgG, and in the second PCR, the reverse primer was MOv18VH_R. For the variable regions in the MOv18 light chain, the cycles for amplification were: 1 min at 98°C, 36 cycles of 10 s at 98°C, 15 s at 57°C, and 15 s at 72°C, followed by a final step at 72°C for 1 min. In the first PCR, the reverse primer was Kappa_R in the constant region of the kappa chain, and in the second PCR, the reverse primer was MOv18K_R. Primers: MOv18VH_F: CAGGTTCAACTGCAGCAGTCTGGA; MOv18VH_R: TGAGGAGACGGTGACTGAGGTTCC; MOv18VK_F: GATATCCAGATGACACAGACTACA; MOv18VK_R: TTTCCAGCTTGGTGCCTCC; IgGRev: CCAACTCTCTTGTCCACCTTGG; Kappa_R: GTTTCTCGTAGTCTGCTTTGCTCA. Forward and reverse primers used for the amplification of unknown Ig light and heavy chain fragments (Table 1), and the nested PCR protocol were previously described (28). PCR products were separated on agarose gels, purified using the QIAquick gel extraction kit (Qiagen), and cloned using the ZeroBlunt PCR cloning kit (Life Technologies). Six colonies were grown for each PCR band, plasmid DNA purified using QIAprep spin miniprep kit (Qiagen) and sequenced by Source Bioscience using the M13 forward primer. Sequences were analyzed using IMGT/V-QUEST (29).

**Table 1:** Nested PCR immunoglobulin primers.

Primer name	Sequence 5′ → 3′	Amplified fragment
VH1L	CCATGGACTGGACCTGGA	Heavy chain
VH2L	CAGATGGACATACTTTGTTCCAC	Heavy chain
VH3L	CCATGGAGTTTGGGCTGAGC	Heavy chain
VH4L	CGATGAAACACCTGTGGTTCTT	Heavy chain
VH5L	ATGGGGTCAACCGCCATCCT	Heavy chain
VH6L	GATGTCTGTCTCCTTCCTCAT	Heavy chain
VH1F	CAGGTGCAGCTGGTGCAGTCTG	Heavy chain
VH2F	GTCTTGTCCCAGGTCAACTTAAGGGAGTCTT	Heavy chain
VH3F	GAGGTGCAGCTGGTGGAGTCTG	Heavy chain
VH4F	CAGGTGCAGCTGCAGGAGTCGG	Heavy chain
VH5F	GAGGTGCAGCTGCTGCAGTCTG	Heavy chain
VH6F	CTGTCACAGGTACAGCTGCAGCAGTCAG	Heavy chain
IGREV	CCAACTCTCTTGTCCACCTTGG	Heavy chain
L-Vk1/2	ATGAGGGTCCCCGCTCAGCTGCTGG	Kappa light chain
L-Vk3	CTCTTCCTCCTGCTACTCTGGCTCCCAG	Kappa light chain
L-Vk4	ATTTCTCTGTTGCTCTGGATCTCTG	Kappa light chain
Ck 543-566	GTTTCTCGTAGTCTGCTTTGCTCA	Kappa light chain
Pan-Vk	ATGACCCAGACTCCATCCACCCTG	Kappa light chain
Ck 494-516	GTGCTGTCCTTGCTGTCCTGCT	Kappa light chain
L-Vλ1	GGTCCTGGGCCCAGTCTGTGCTG	Lambda light chain
L-Vλ12	GGTCCTGGGCCCAGTCTGCCCTG	Lambda light chain
L-Vλ3	GCTCTGTGACCTCCTATGAGCTG	Lambda light chain
L-Vλ4/5	GGTCTCTCTCGCAGCCTGTGCTG	Lambda light chain
L-Vλ6	GTTCTTGGGCCAATTTTATGCTG	Lambda light chain
L-Vλ7	GGTCCAATTCCCAGGCTGTGGTG	Lambda light chain
L-Vλ8	GAGTGGATTCTCAGACTGTGGTG	Lambda light chain
Cλ-1	CACCAGTGTGGCCTTGTTGGCTTG	Lambda light chain
F-Vλ1	CAGTCTGTGTTGACGCAGCC	Lambda light chain
F-Vλ2	CAGTCTGCCCTGACTCAGCC	Lambda light chain
F-Vλa3	TCTTATGAGCTGACACAGCCA	Lambda light chain
F-Vλa3l	TCTTCTGAGCTGACTCAGGACCC	Lambda light chain
F-Vλ4ab	GAGCTTGTGCTGACTCAATC	Lambda light chain
F-Vλ4c	CTGCCTGTGCTGACTCAGC	Lambda light chain
F-Vλ5/9	CAGCCTGTGCTGACTCAGCC	Lambda light chain
F-Vλa6	AATTTTATGCTGACTCAGCCCCACT	Lambda light chain
F-Vλ7/8	CAGACTGTGGTGACCCAGGAG	Lambda light chain
F-Vλa10	CAGGCAGGGCTGACTCAG	Lambda light chain
Cλ-2	GGGTGGGAACAGAGTGACC	Lambda light chain

### Recombinant Antibody Cloning and Expression

Heavy and light chain variable regions were cloned in pVitro-hygro-1 (Invivogen) using polymerase incomplete primer extension (PIPE) cloning as previously described (25). In brief, pVitro-hygro-1, containing pre-cloned human antibody heavy and light constant region cassettes (gamma 1/kappa) was used as a template in two separate PIPE PCR reactions to amplify two linear plasmid fragments with partially single-stranded 5″ ends. Similarly, the Ig heavy and light chain variable regions were PIPE PCR-amplified, using the pCR-Blunt constructs, generated previously, as PCR templates. Next, the PCR products were diluted four times with ddH_2_O and mixed in a ratio of 1:1:1:1, incubated at RT for 1 h, and 10 µl of the mixture were used to transform Top10 OneShot™ *E. coli* cells (Thermo Fisher Scientific). Successful cloning was confirmed by Sanger sequencing (Source BioScience). MOv18 IgG1/k was expressed transiently in Expi293F™ cells (Thermo Fisher Scientific), as described in Ref. (26). Antibodies, secreted in the Expi293F™ culture supernatant, were purified using a Protein A column (Thermo Fisher Scientific) and stored in PBS at 4°C.

## Results

### Workflow for Identification of Antibody-Expressing Single Cells Using Antigen-Conjugated Fluorescent Beads

**Figure 1:**
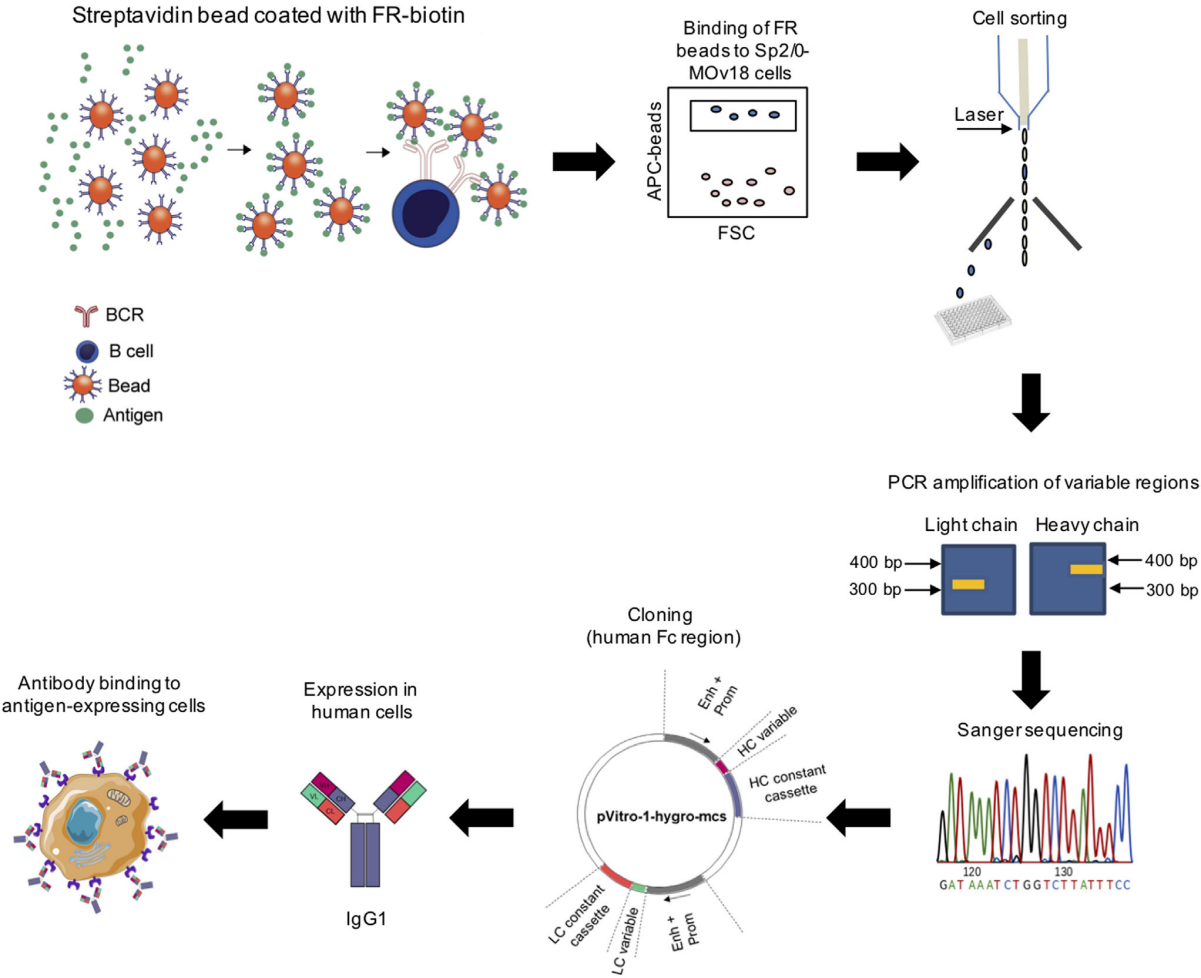
Workflow for identification of antigen-specific monoclonal antibodies derived from single cell cloning. Biotinylated antigen (Folate Receptor alpha, FRα) was conjugated to fluorescent beads conjugated to streptavidin or avidin. Antigen-coated beads could bind cells expressing an antigen-specific immunoglobulin (Ig) and bead-bound cells were purified using cell sorting. RNA from sorted cells was reverse transcribed and Ig variable regions were amplified by nested PCR, sequenced, and cloned using the dual expression plasmid pVITRO1 containing human heavy and light chain constant region cassettes (gamma1/kappa). Antibodies were expressed in a human cell expression system, purified, and tested for antigen binding.

We designed a process to allow the identification of antigen-specific antibody-expressing B cells, which can be performed without prior *ex vivo* growth or secondary screening of B cells. To establish this system, we selected Folate Receptor alpha (FRα) as a model antigen. As the test antibody-expressing cells, we selected a B cell line expressing both the soluble and the membrane-bound [B cell receptor (BCR)] form of a human/mouse chimeric antibody (MOv18 IgG1 clone) specific for FRα. The workflow (Figure 1) entails coupling of fluorescent polystyrene beads with the nominal antigen, Folate Receptor alpha (FRα), followed by binding of FRα-coated beads to anti-FRα antibody-expressing B cells. Single bead-conjugated cells were identified and isolated by FACS sorting directly onto microplates containing lysis buffer. RNA released from single cells was converted to cDNA using reverse transcription followed by a semi-nested RT-PCR with Ig specific primers. Matched variable H and L chains were sequenced and cloned into a single expression vector containing the H and L constant IgG1 region sequences as previously reported (25, 26). The full antibody was then expressed in a human expression host. The antibody was purified and antibody specificity for the antigen was tested on target antigen-expressing cells.

### Antigen-Coupled Fluorescent Bead Recognition by Monoclonal Antibodies and by Specific Antibody-Expressing B Cells

**Figure 2:**
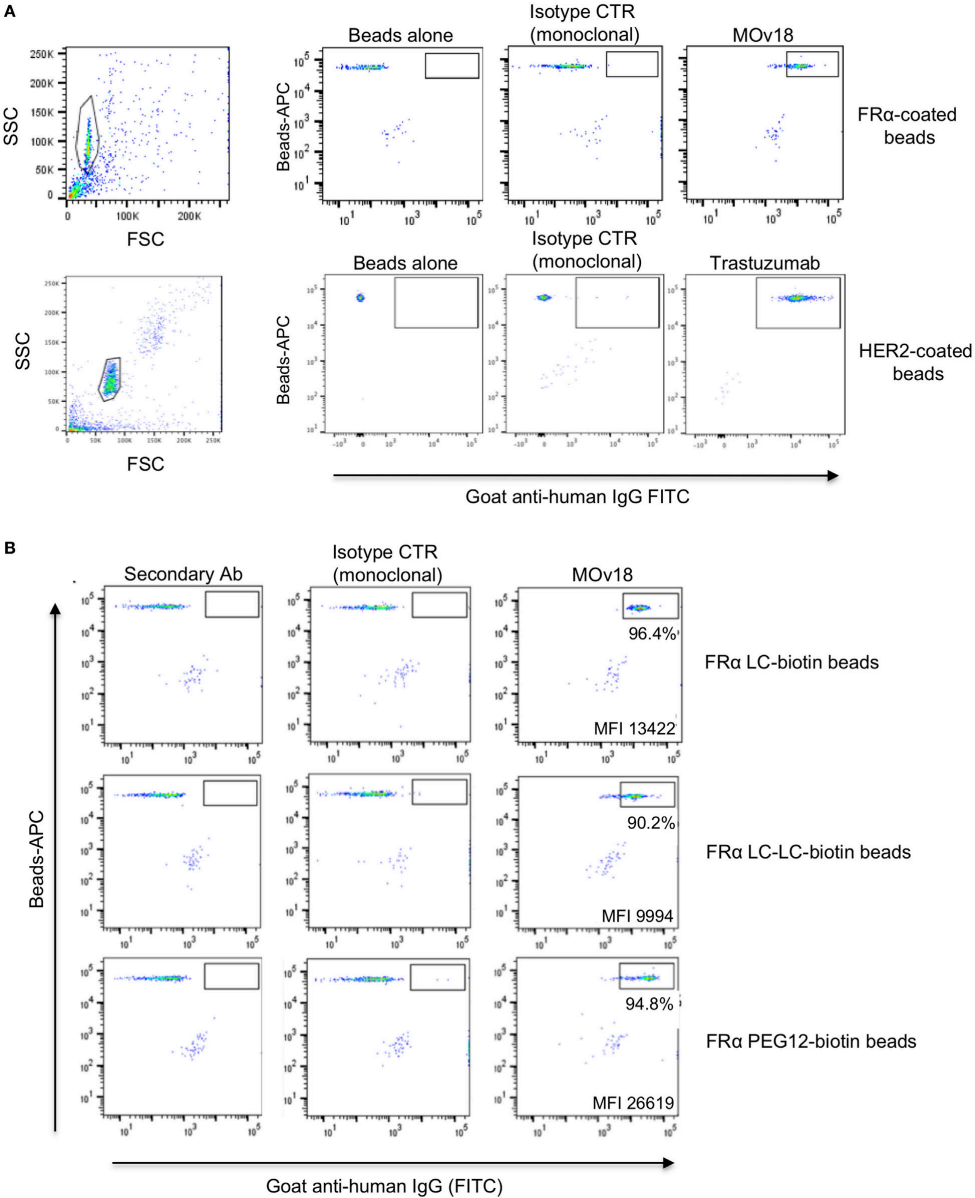
Antigen-coated beads were recognized by specific antibodies. **(A)** Fluorescent microspheres (SA-Blue 1.1 µm) coated with human recombinant FRα (top) or HER2 (bottom) conjugated to LC-LC-biotin were incubated with no antibody, non-specific monoclonal anti-NIP (control), MOv18 (top right), or anti-HER2 antibodies (bottom right). Bead-bound antibodies were detected using secondary goat anti-human FITC-conjugated antibodies. Gates indicate positive antibody staining above controls. **(B)** Recombinant FRα conjugated to biotin of different arm lengths was used to coat fluorescent LumAvidin 5.6 µm microspheres, and microspheres were stained with MOv18 IgG1 antibody. The percentage of MOv18 IgG1-bound beads and mean fluorescence intensity (MFI) values of positive populations are indicated in each dot plot.

We first established the workflow using APC fluorochrome LumAvidin Microspheres of 5.6 µm diameter (LumAvidin 5.6 µm; Table S1 in Supplementary Material). Prior to interrogating the ability of these beads to bind antibody-expressing cells, we used flow cytometric evaluations to confirm that fluorescent beads could be successfully coupled to antigens. We tested the antigens FRα and HER2 conjugated to fluorescent beads. The anti-FRα antibody MOv18 IgG bound to FRα-conjugated beads, and the HER2-specific antibody trastuzumab bound to HER-2-conjugated beads. On the other hand, the hapten-specific monoclonal antibody NIP IgG showed only background binding to FRα-conjugated or to HER-2-conjugated beads, and no binding was detected on beads incubated with secondary antibody alone (Figure 2A). We also interrogated different coupling agents with varying biotin lengths (LC-biotin, 22.4 Å; LC-LC-biotin, 30.5 Å; PEG12-biotin, 56 Å) to assess whether the distance between bead and antigen from the bead surface could affect antigen recognition by anti-FRα-expressing B cells. We found no significant differences in MOv18 IgG antibody binding to bead-conjugated FRα with respect to any of these coupling agents (Figure 2B).

**Figure 3:**
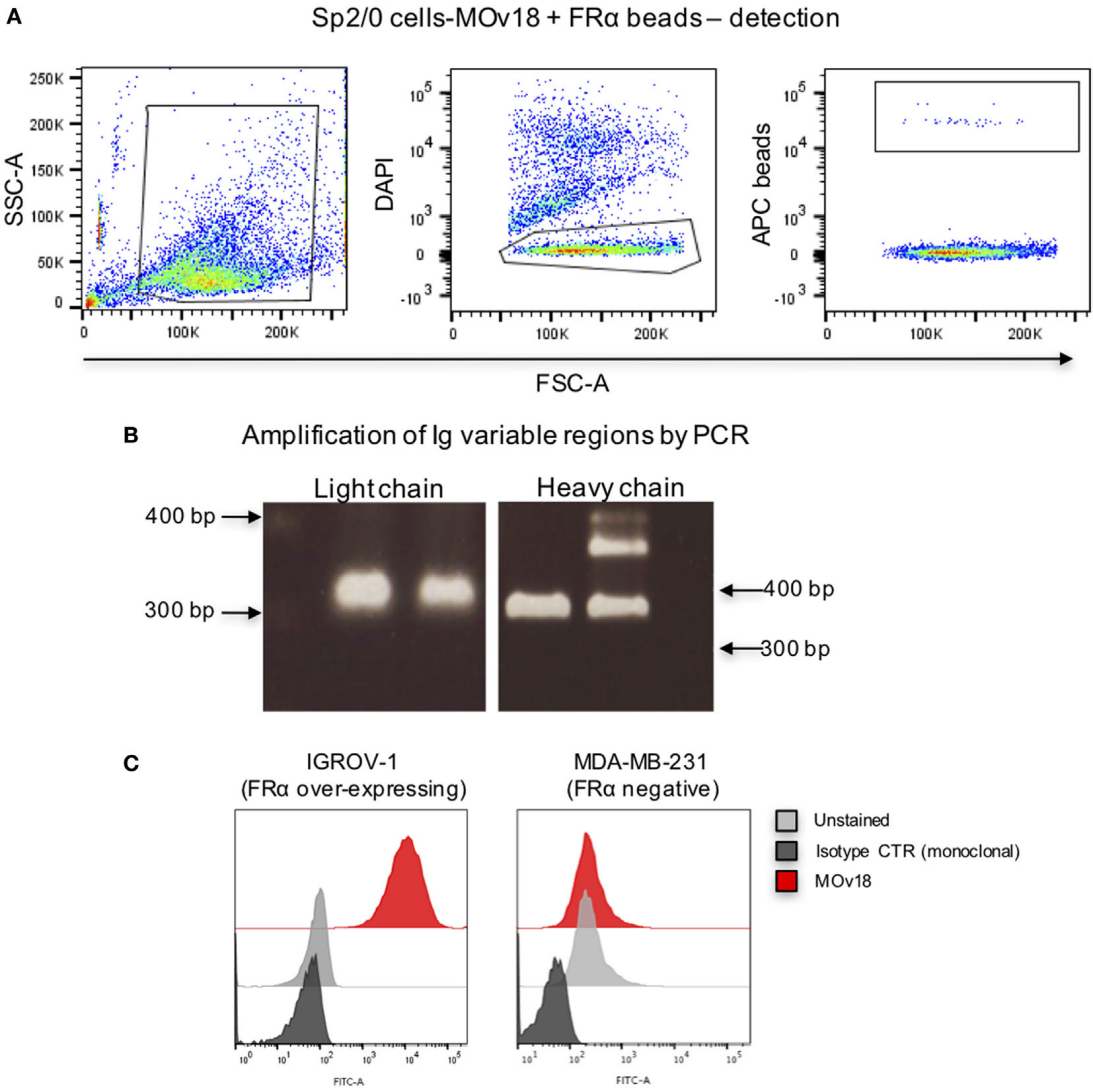
Binding of folate receptor α+ beads to B cells expressing MOv18 antibody. **(A)** Gating strategy for Sp2/0 cells (10^5^ in 100 µl FACS buffer) expressing MOv18 IgG, incubated with fluorescent LumAvidin 5.6 µm microspheres coated with FRα *via* PEG12-biotin. Single live cells binding to the beads were sorted into individual wells in a 96-well plate. **(B)** Agarose electrophoresis of the heavy and light chain variable regions of immunoglobulin sequences obtained from the Sp2/0 cells and amplified by PCR. **(C)** Flow cytometric evaluations of purified MOv18 antibody, produced in-house from single Sp2/0 cell Ig sequences, and expressed as a human/chimeric IgG1. MOv18 recognized FRα-overexpressing IGROV1 cancer cells but not FRα-negative MDA-MB-231 breast cancer cells. Dark gray histogram: unstained IGROV1 cells; light gray histogram: non-specific anti-NIP (monoclonal) antibody alone; Red histogram: MOv18 antibody cloned from a single B cell.

We then established a model system using microsphere-coupled FRα and anti-FRα antibody-expressing B cells. We confirmed that a small proportion of FRα+ LumAvidin 5.6 µm microspheres recognized single anti-FRα antibody-expressing B cells (Sp2/0-MOv18) (Figure 3A). When single bead-bound cells were isolated into lysis buffer-containing microplate wells by flow cytometric sorting, single cell PCR yielded matched H and L chain variable region DNA products were detected to be of the expected size (Figure 3B). Antibody variable region sequences were cloned into a pVITRO1 vector containing human IgG1/k constant region cassettes and the antibody was expressed in Expi293F™ cells. Subsequently, the cloned IgG1 antibody was used to assess binding to IGROV1 cells, which express native cell surface human FRα. Flow cytometric evaluations confirmed that the anti-FRα antibody cloned from a sorted single antibody-expressing B cell was able to specifically recognize antigen-expressing IGROV1 cells. The cloned anti-FRα antibody did not recognize MDA-MB-231 breast carcinoma cells that do not express FRα (Figure 3C).

These findings confirmed that it is possible to extract matched variable region sequences from single antibody-expressing cells selected by recognition of specific antigen-coated microspheres. Selection of single B cells allowed the identification of the expected matched variable regions and permitted the cloning and production of the corresponding monoclonal antibody from a single cell. This antibody was able to recognize natively expressed cell surface FRα.

### Influence of Microsphere Diameter and Biotin Length on Specific Recognition of Antigen-Expressing B Cells

Since we observed that FRα+ microspheres were able to bind only a small proportion of single anti-FRα antibody-expressing B cells (Sp2/0-MOv18), we investigated whether microsphere diameter or the FRα-biotin on the beads could influence specific recognition of antibody-expressing cells.

**Figure 4:**
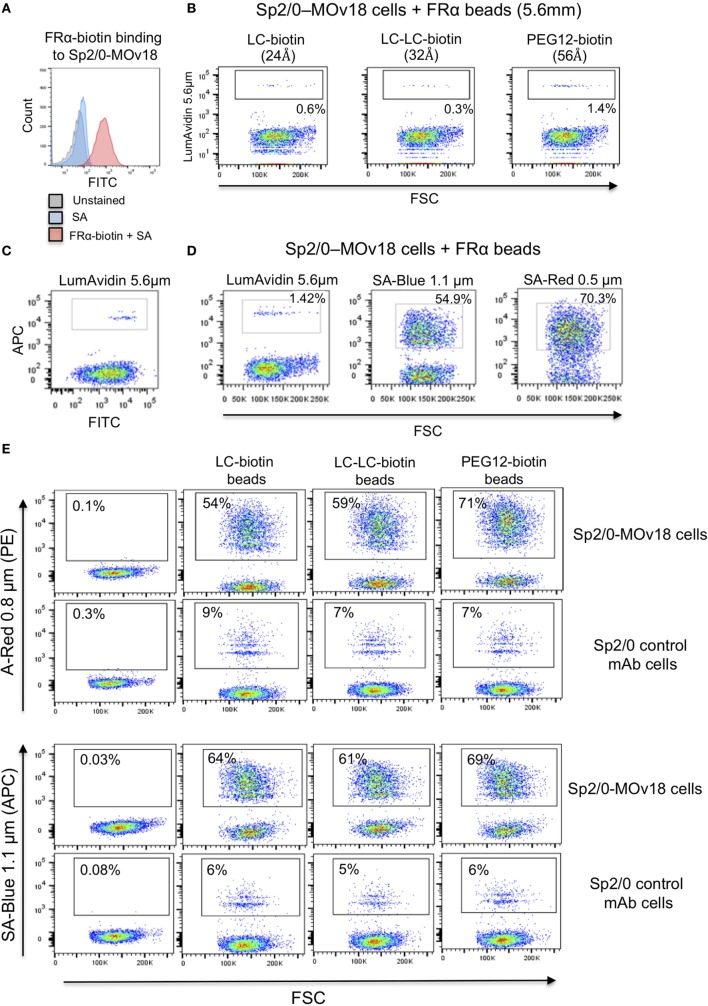
The size of the fluorescent beads conjugated to FRα affects binding of MOv18 IgG-expressing cells. **(A)** Sp2/0-MOv18 IgG cells stained with FRα-biotin confirm expression of MOv18 IgG on the cell surface. Gray histogram: unstained cells; Blue histogram: cells given Streptavidin_AF488 alone; Red histogram: cells stained with FRα-biotin and Streptavidin_AF488. **(B)** Flow cytometric analyses of the effects of the biotin arm lengths on fluorescent FRα-coated LumAvidin 5.6 µm bead binding to Sp2/0-MOv18 cells. **(C)** Sp2/0-MOv18 cell binding to FRα-coated LumAvidin 5.6 µm beads was confirmed with an anti-human IgG antibody. This showed binding of beads to the Sp2/0 cells expressing higher levels of anti- FRα antibody. **(D)** Flow cytometric analyses of the effects of fluorescent FRα-coated bead size (LumAvidin 5.6 µm, SA-Blue 1.1 µm, SA-Red 0.5 µm) on binding to Sp2/0-MOv18 cells. **(E)** Effects of length of the biotin arm on binding of A-Red 0.8 µm and SA-Blue 1.1 µm beads to Sp2/0-MOv18 cells (left panels: cells alone).

We confirmed that Sp2/0-MOv18 IgG cells expressing MOv18 IgG on the cell surface could be recognized by human recombinant FRα (Figure 4A). As observed with recombinant antibody binding to FRα-coated microspheres (Figure 2), the distance between bead and antigen from the bead surface did not affect antigen recognition by anti-FRα antibody-expressing Sp2/0-MOv18 cells (Figure 4B). However, the proportion of the Sp2/0-MOv18 cells recognized by fluorescent beads coated with FRα differed with bead size or type: microspheres of smaller bead diameters (SA-Red 0.5 µm and SA-Blue 1.1 µm) appeared to bind a higher proportion of B cells (70.3 and 54.9%, respectively) compared to the LumAvidin 5.6 µm beads (1.4%) (Figures 4C,D).

The length of the biotin arm had minor effects on the binding of the smaller sized (A-Red 0.8 µm and SA-Blue 1.1 µm) microspheres to Sp2/0-MOv18 cells. The PEG12-biotin arm showed a slightly higher proportion of microsphere-attached Sp2/0-MOv18 cells (69–71% of cells) compared with beads attached to FRα *via* LC-LC-biotin (59–61%) or LC-biotin (54–64%) arms (Figure 4E). Binding of FRα-coated fluorescent beads appeared to be specific for antibody-expressing B cells, since FRα-coated beads bound 54–71% of Sp2/0-MOv18 IgG cells, while FRα-coated beads bound 6–9% of Sp2/0 non-specific antibody-expressing cells used as controls (Figure 4E).

These findings suggest that bead size or type could influence the proportion of possible antibody-expressing B cells recognized by antigen-conjugated fluorescent beads.

### Evaluation of Specific Detection of Antibody-Expressing B Cells in PBMC Samples by Antigen-Conjugated Fluorescent Beads

**Figure 5:**
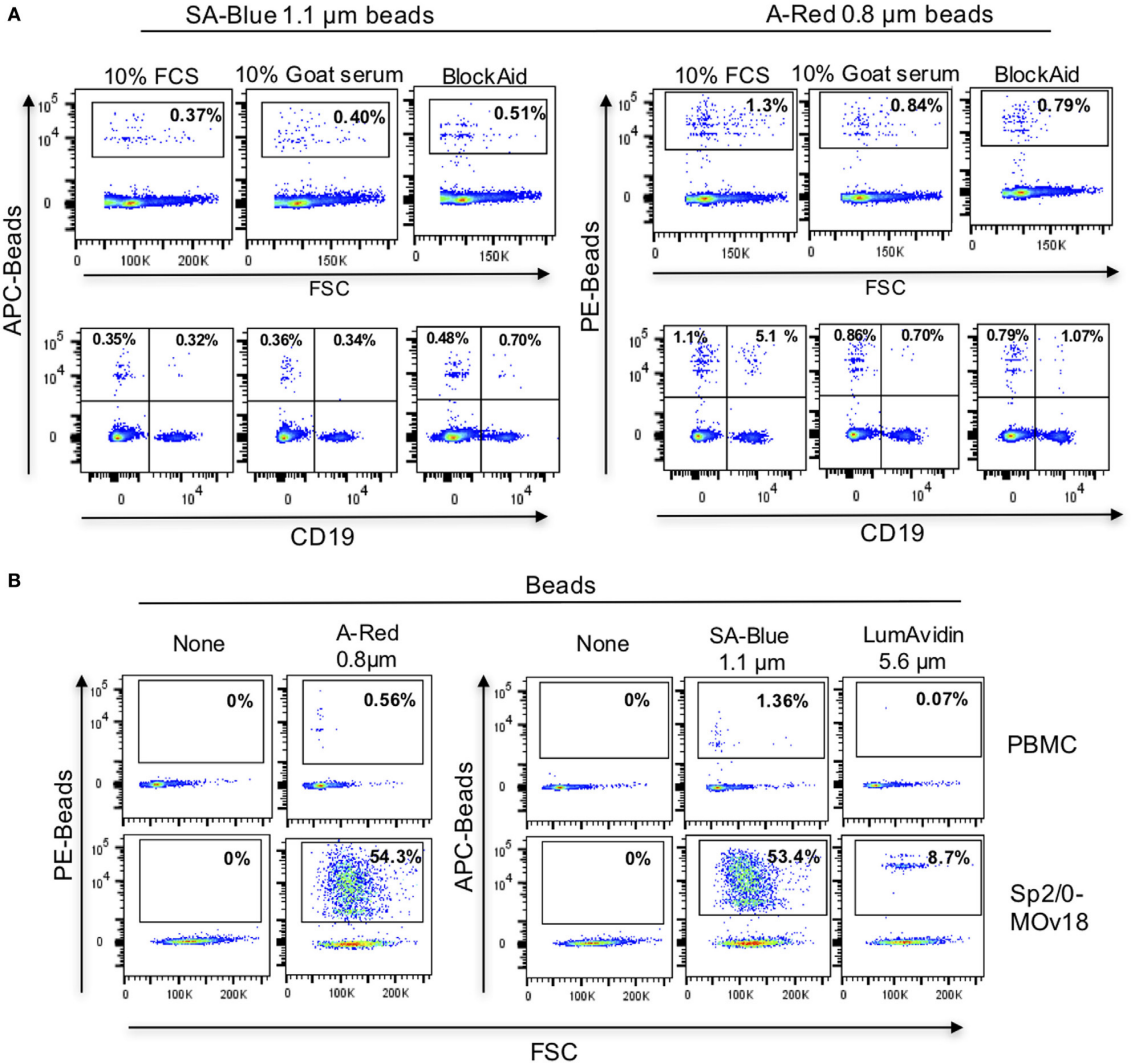
Background binding of fluorescent beads to human peripheral blood mononuclear cells (PBMC). **(A)** Flow cytometric analyses demonstrating the effects of blocking agents in reducing the background binding of the SA-Blue 1.1 µm and A-Red 0.8 µm beads to cells. Top panels: non-specific binding to total PBMC; Bottom panels: background among B cells. **(B)** Analyses of FRα-coated A-Red 0.8 µm, SA-Blue 1.1 µm, and LumAvidin 5.6 µm beads incubated with PBMCs (to establish the background staining of the beads), or with Sp2/0-MOv18 cells (to establish binding to antigen-reactive positive cells).

Since alongside specific binding of beads to antibody-expressing B cells, we also detected non-specific binding of FRα-coated beads to control Sp2/0 cells (Figure 4E), we further investigated the background binding of fluorescent beads to freshly isolated human PBMCs. A proportion (~1%) of human PBMCs (top panel) and also of human B cells (CD19+ cells, lower panel) could bind to FRα-coated beads in most likely a non-specific manner (Figure 5A). Different blocking agents did not appear to reduce the levels of background binding (Table S2 in Supplementary Material). Furthermore, A-Red 0.8 µm, SA-Blue 1.1 µm, and LumAvidin 5.6 µm FRα-coupled (PEG12-biotin) beads were incubated with PBMCs and binding was directly compared to recognition of Sp2/0-MOv18 IgG cells (used as positive controls). FRα-coupled A-Red 0.8 µm microspheres showed 54.3% specific and 0.56% non-specific B cell recognition, while SA-Blue 1.1 µm microspheres showed 53.4% specific and 1.36% non-specific recognition of B cells. On the other hand, the larger sized LumAvidin 5.6 µm microspheres showed 8.7% specific and 0.07% non-specific binding (Figure 5B). This suggested that the smaller diameter beads were more likely to select antibody-expressing cells.

**Figure 6:**
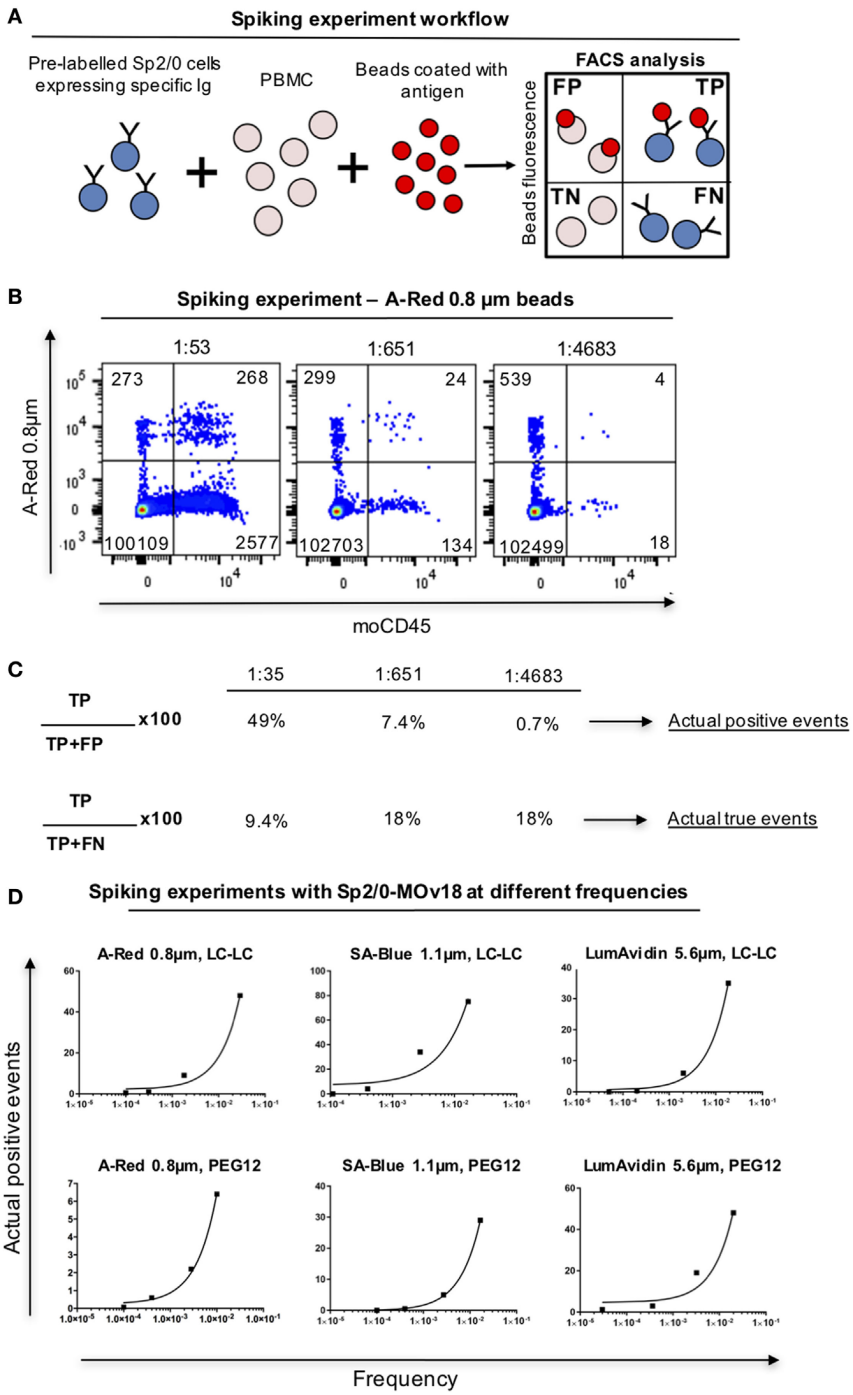
Detection of antibody-expressing B cells in peripheral blood mononuclear cells (PBMC) samples using fluorescent beads. **(A)** Schematic representation of Sp2/0 “spiking” of PBMCs. Flow cytometric dot plots are separated in quadrants labeled as follows: FP, false positive; TP, true positive; TN, true negative; FN, false negative. **(B)** Flow cytometric analyses of A-Red 0.8 µm beads incubated with PBMC spiked with different numbers of Sp2/0-MOv18 cells pre-labeled with an anti-CD45 antibody. Actual dilutions are indicated above each dot plot. The actual dilution was calculated by dividing the number of live Far-red positive events (antigen specific cells) by the number of live Far-red negative events (PBMC). Numbers in each quadrant indicate the absolute number of cells detected. **(C)** Calculation of Actual positive events and Actual true events from data obtained with “spiking” of PBMCs. Actual positive events and Actual true events for each frequency tested in B were calculated using the formulas on the left. **(D)** FRα+ beads of different sizes recognize Sp2/0-MOv18 B cells found at different frequencies in PBMCs. Actual positive events out of all bead-bound events were analyzed and demonstrate correlation between cell frequency and percentage of Actual positive events. A-Red 0.8 µm beads (left); SA-Blue 1.1 µm beads (middle), and LumAvidin 5.6 µm beads (right). Top panels: LC-LC biotin coupling; bottom panels: PEG12 biotin coupling.

In order to analyze whether, and at what frequency, anti-FRα antibody-expressing B cells in PBMC samples may be detected by FRα-coupled beads, we spiked human PBMC with Sp2/0-MOv18 cells at different frequencies and we detected double-positive (FRα+/Sp2/0+) events by flow cytometry (Figure 6A). FRα-coupled A-Red 0.8 µm fluorescent beads were incubated with PBMC (1 µl beads/10^6^ PBMC, Table S1 in Supplementary Material) in the presence of serially diluted (1:50, 1:500, 1:5,000) Sp2/0-MOv18 cells pre-labeled with an anti-CD45 antibody. The actual dilution was calculated post acquisition (Figure 6B). Actual positive events were defined as anti-FRα antibody-expressing B cells among all bead-bound cells and used to evaluate potential specific selection of real antigen-specific cells. The Actual true events were defined as antigen-specific B cells bound to beads among all possible antibody-specific Sp2/0+ events and used to estimate the selection of antigen-binding cells compared to all specific cells in a sample. We observed that FRα+ beads were able to identify 49% actual positive events (B cells) when the specific B cell frequency was ~1:50. The proportion of actual positive events (anti-FRα antibody-expressing B cells) was lower when the specific B cell frequencies in the PBMC pool were reduced (Figures 6B,C). The actual true events (B cells bound to beads among all possible antibody-expressing Sp2/0+ events) ranged between 9.4 and 18% (Figures 6B,C). Beads of different types and diameter sizes varied in ability to detect Actual positive events, and the proportion of Actual positive events (among all bead-bound cells) decreased when the frequencies of FRα+/Sp2/0+ B cells were lower among PBMCs. Between 5 and 75% of antibody-expressing B cells recognized by beads were antigen-specific when B cells were found in high frequencies (≥1:100) in human blood. SA-Blue 1.1 µm LC-LC biotin beads detected the highest proportion (75%) when B cells were found at a frequency of 1:43 (Figure 6D; Table S3 in Supplementary Material).

**Figure 7:**
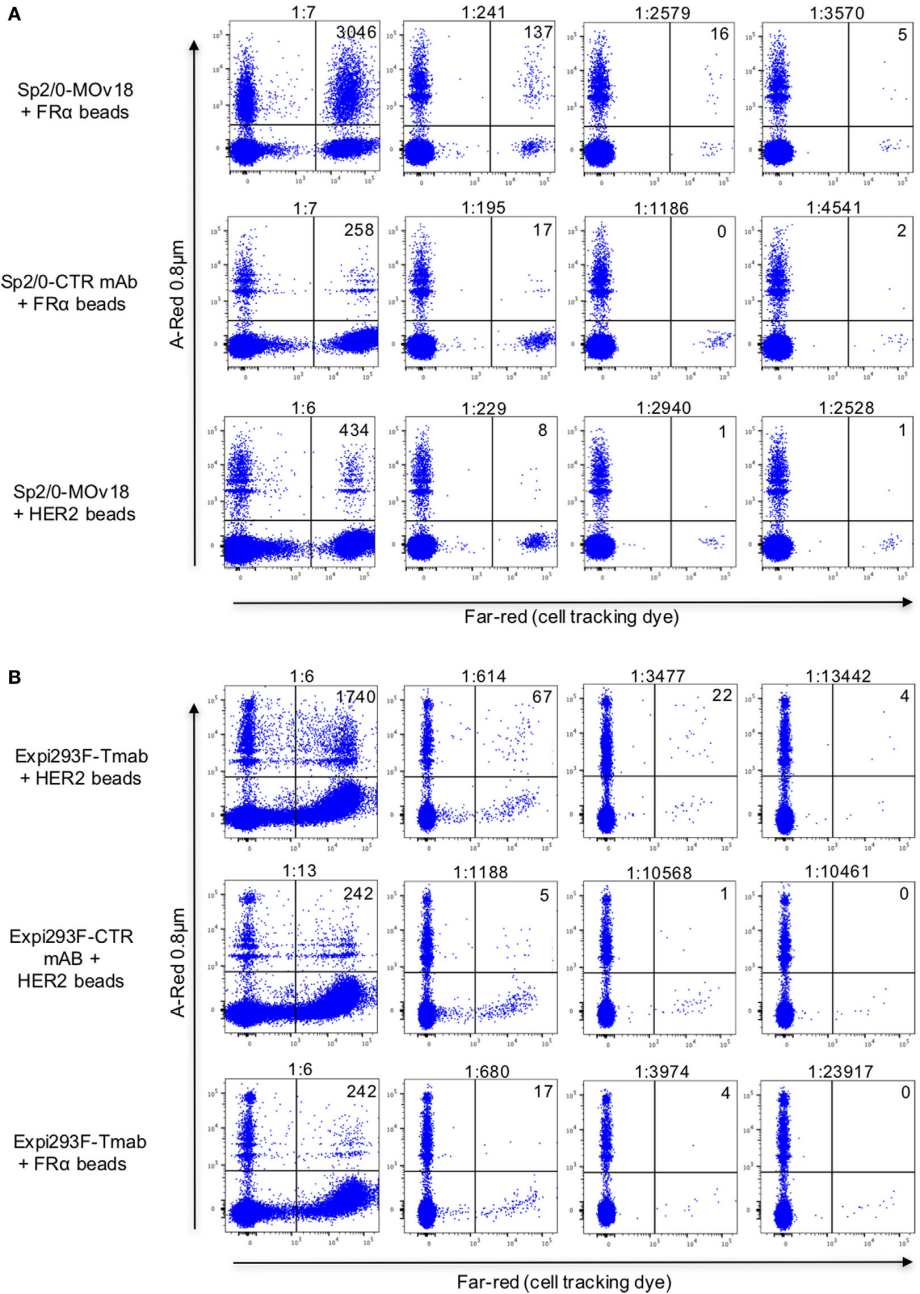
Detection of antibody-expressing or non-specific control antibody-expressing cells in peripheral blood mononuclear cells (PBMC) samples using fluorescent beads conjugated to recombinant antigens. **(A)** Flow cytometric dot plots of human PBMC incubated with FRα-coated A-Red 0.8 µm beads (top and middle) or with recombinant HER2-coated beads (bottom), spiked with different numbers of either Sp2/0-MOv18 cells (top and bottom), or non-specific SF25 control monoclonal antibody-expressing Sp2/0 cells (middle), pre-labeled with Far-red cell tracking dye. Actual dilutions (calculated post acquisition) are indicated above each dot plot. The actual dilution was calculated by dividing the number of live Far-red positive events (antigen specific cells) by the number of live Far-red negative events (PBMC). Numbers in each quadrant indicate the absolute number of bead-bound cells detected. **(B)** Flow cytometric analyses of human PBMC incubated with A-Red 0.8 µm beads coated with human recombinant HER2 (top and middle) or with FRα (bottom), and spiked with different numbers of Expi293F cells expressing an anti-HER2 antibody (trastuzumab, top and bottom) or non-specific monoclonal antibody control (anti-CSPG4, middle). Expi293F cells were pre-labeled with Far-red cell tracking dye. Numbers in each quadrant indicate the absolute number of cells detected by antigen-coated beads.

To confirm selection of antibody-expressing cells by specific antibody, PBMC spiking showed that FRα-coated beads were more likely than HER2-coated beads to be recognized by FRα-specific Sp2/0-MOv18 cells (Figure 7A; Figure S1 in Supplementary Material). Anti-SF25 antibody-expressing Sp2/0 cells were also less likely to be detected by FRα-coated beads in PBMC samples (Figure 7A). Similarly, HER2-coated beads were more likely to bind anti-HER2 (trastuzumab)-expressing Expi293F cells compared with anti-CSPG4 control antibody-expressing Expi293F cells. HER2-coated beads were also more likely to bind anti-HER2 (trastuzumab)-expressing Expi293F cells compared with FRα-coated beads, which showed low binding to anti-HER2 (trastuzumab)-expressing Expi293F cells (Figure 7B). Human Ig expression on the surface of Expi293F cells is shown in Figure S1 in Supplementary Material. Together, these data suggest that antibody-expressing cells in PBMC preparations are able to recognize beads conjugated to their specific antigens.

**Figure 8:**
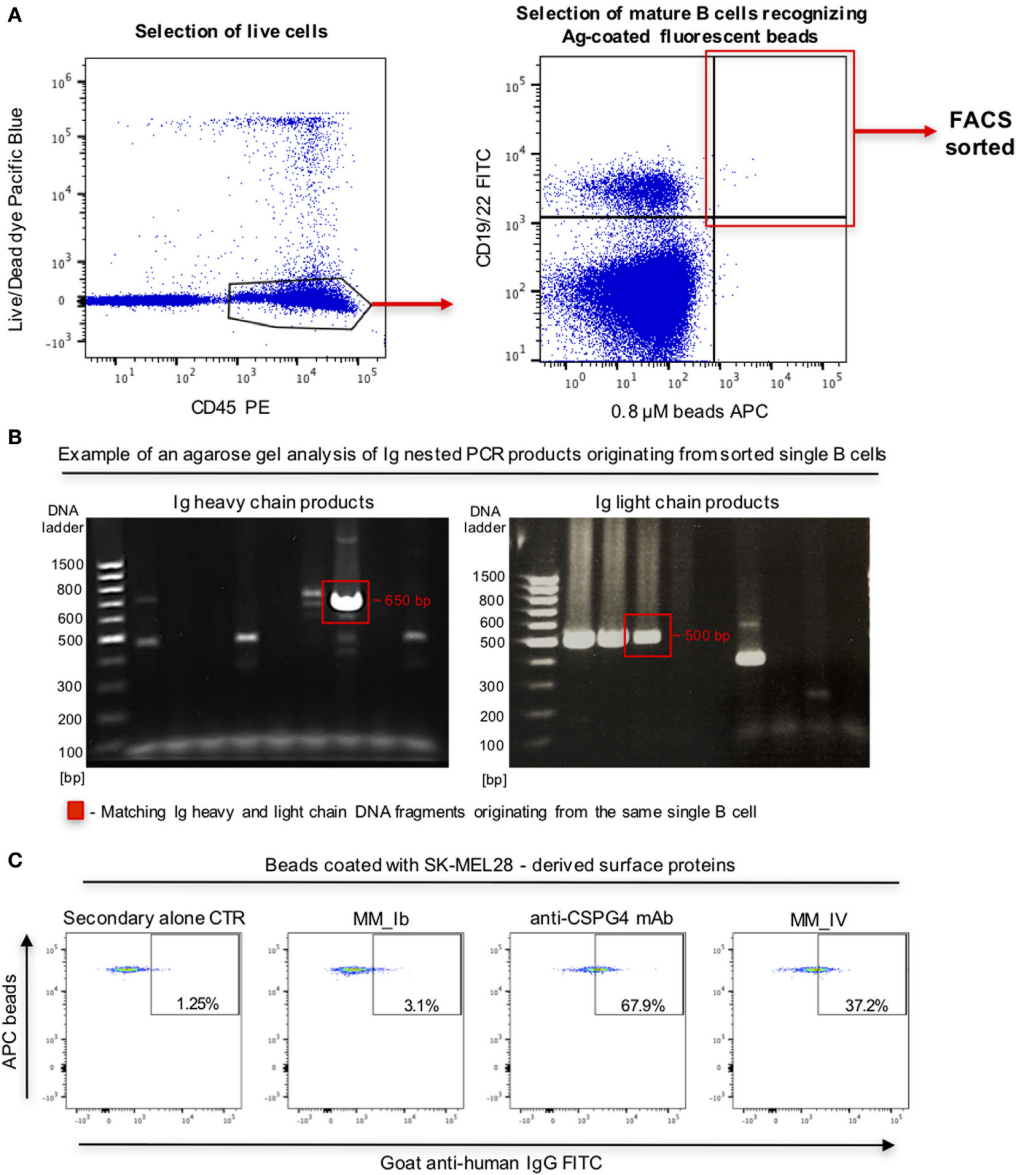
Selection of antibody-expressing B cells in peripheral blood mononuclear cells (PBMC) samples from patients with melanoma. **(A)** Representative flow cytometric dot plots of cell sorting gating strategy to select melanoma patient B cells recognizing melanoma cell line surface antigen (Ag)-coated fluorescent beads. Viable (live/dead dye) human CD45+ PBMC incubated with antigen-coated beads were identified (left) and B cells (CD19/CD22+ -FITC) recognizing antigen-coated beads (APC) were selected for single cell sorting (right). **(B)** Agarose gels of Ig heavy (left) and light (right) chain DNA fragments amplified through nested PCR. Each gel lane represents PCR products derived from a single B cell. Fragments of the expected sizes (~650 bp for the heavy chain, ~500 bp for the light chain fragments) were extracted and sequenced. Red boxes indicate matching Ig heavy and light chain DNA fragments originating from the same single B cell. **(C)** Flow cytometric dot plots depicting binding of two single B cell-derived monoclonal antibody clones (MM_Ib, MM_IV) to melanoma cell antigen-coated fluorescent beads (SK-MEL-28 melanoma cells). The bead-selected clone (MM_IV) bound to antigen-coated beads while the non-specific one (MM_Ib) did not show reactivity. Background binding was detected with secondary antibody alone, while beads were recognized by the positive control anti-CSPG4 antibody.

To interrogate this approach in the human setting, we evaluated whether fluorescent beads conjugated to melanoma cell surface antigens were able to identify antigen-reactive B cells. We prepared fluorescent beads coated with antigens extracted from human melanoma SK-MEL-28 cells, which natively express the tumor-associated antigen chondroitin sulfate proteoglycan 4 (CSPG4). Melanoma antigen-coated beads were recognized by an anti-CSPG4 antibody but not by MOv18 antibody specific for the antigen FRα not expressed by SK-MEL-28 cells (Figure S2 in Supplementary Material), suggesting that antigen-reactive antibodies can specifically recognize antigen bound on these beads. We then screened for antigen-reactive antibodies from human B cells. PBMCs from patients with melanoma incubated with melanoma antigen-coated beads were screened to identify mature CD19/CD22+ B cells recognizing antigen-coated beads. Single bead-bound B cells were isolated by single cell sorting Ig heavy and light chain sequences were amplified, sequenced, and antibodies were cloned (Figures 8A,B). An antibody derived from a single bead-bound B cell could recognize melanoma antigen-coated fluorescent beads compared with secondary only or a non-specific antibody control. The B cell-derived clone showed binding comparable to that of a monoclonal antibody specific for the melanoma-associated antigen CSPG4 (Figure 8C).

Taken together, these findings suggest that antigen-reactive B cells in human blood could be recognized by antigen-conjugated fluorescent beads and could be sorted by flow cytometry for the expression of monoclonal antibodies.

## Discussion

In this report, we present the development of a fluorescent bead method for the selection of single antigen-reactive B cell clones and the production of the cloned antigen-specific antibodies for downstream testing. The workflow comprises: (a) conjugation of fluorescent microspheres with a recombinant antigen of interest; (b) identification of fluorescently labeled single antibody-expressing B cells by flow cytometry using antigen-conjugated beads; (c) flow cytometric sorting of bead-bound single cells directly into lysis buffer; (d) single cell retrotranscription and sequencing of matched heavy and light chain antibody variable regions; (e) cloning and expression using a vector containing human constant region cassettes; (f) confirmation of antigen reactivity of the produced full-length antibody. We designed this process to allow the identification of antigen-specific antibody-expressing B cells without the requirement of prior *ex vivo* growth or secondary screening of B cells, and we aimed to conduct the workflow from clone identification to antibody production and characterization in a timeframe of approximately 23 days.

To design this protocol, we employed a model system featuring human recombinant Folate Receptor alpha (FRα) as the target antigen, a B cell line expressing both the soluble and the membrane-bound forms of MOv18 IgG1, a human/mouse chimeric antibody specific for FRα. We showed that fluorescent microspheres can be coupled with the recombinant antigen *via* biotin–streptavidin/avidin bridging, and that immobilized antigens could be readily detected by antigen-specific monoclonal antibodies. Recognition of the epitope on FRα by the test antibody MOv18 is thought to be dependent on the native folding of the target antigen. Here, we demonstrated that it is possible to use antigen-coupled fluorescent beads of different sizes to identify and isolate single B cells expressing cell surface-expressed antibodies that recognize this conformational epitope. Antigen-antibody recognition could be improved by evaluating beads with different characteristics and diameters, while varying the lengths of the avidin coupling agents did not significantly influence antibody-expressing cell recognition by the beads. The latter observation also suggested that engagement of the antigen to the bead surface *via* different biotins did not mask epitope recognition by specific antibody either in solution or when the antibody was expressed on the surface of a B cell.

A key feature of this streptavidin–biotin bead-based approach is that, in principle, it may enable the coupling of virtually any known native or recombinant antigen to fluorescent beads and facilitate the detection of B cells reactive to this antigen. Our strategy may offer different features compared with those of other detection methods. For instance, antibodies used for detection of BCR on B cells may also interact not only with BCRs but also with Fc receptors on human B cells (30). Tetramer technologies may be applicable to the selection of antigen-specific B cells, but fluorophores have been known to be released from antigen complexes and to bind non-specifically to immune cells, yielding false positive events (31). Our protocol is also specifically designed to avoid the requirement for B cell culture and *ex vivo* expansion or for antibody selection from culture supernatants prior to sub-cloning, limiting dilution and isolation of antigen-specific B cell clones. As well as being laborious and lengthy, these steps may also be limited by specific expansion of distinct B cell populations not always representative of the original B cell repertoire, and which could suffer from potential loss of the antigen-specific clone during the cell *ex vivo* culture processes (17).

Following flow sorting of single cells directly into lysis buffer, we confirmed that it is possible to extract matched H and L chain variable region sequences from single antibody-expressing cells selected by specific antigen-coated beads. Employing the FRα-MOv18 Sp2/0 B cell model, we confirmed that B cell selection by FRα+ fluorescent beads allowed the identification of the matched variable regions and permitted the cloning and production of the corresponding monoclonal antibody from a single cell. This was greatly expedited with the use of a single vector Expi293F™ cell line-based expression system, which permitted antibody production within a few days (25, 26). While the vector used in this study contained the constant regions of human IgG1, in principle, this platform could be used for engineering of B cell-derived antibodies with constant regions of any isotype or species desired, potentially facilitating a wide range of downstream applications for this technology. Importantly, we showed that cloned and expressed full-length antibody with variable regions extracted from a single B cell could recognize native cell surface-expressed human FRα. The specificity of an identified antibody could ultimately be confirmed only by the expression of the antibody and subsequent testing against the natively expressed antigen. Previous published studies report the number of detected B cells and the antigenic reactivity of antibodies secreted in supernatants of *ex vivo* B cell cultures without cloning, production, or testing of the derived antibody clones (16, 32). A similar B cell identification process to the one reported in our study described the frequency of antigen-specific B cells detected using a modified bead-based method (31). With this tool, it was possible to monitor the frequencies of anti-HLA, anti-tetanus toxin-, and anti-EBNA1-committed B cells in different individuals. However, antibodies from selected B cells are often not cloned and expressed subsequently in order to analyze their ability to recognize natively expressed cognate antigens. Our data confirming antibody sequencing, cloning, expression, and antigen recognition provide an early proof of principle that functional Ig sequences could be recovered with this methodology.

We ascertained that binding of FRα-coated fluorescent beads can specifically single out antibody-expressing B cells. We found that FRα-coated beads bound up to 71% of possible Sp2/0-MOv18 IgG cells. However, alongside enhanced recognition of possible antigen-reactive B cells, FRα-coated beads also bound to 6–9% of non-specific Sp2/0 B cells. Furthermore, a proportion (~1%) of human circulating B cells could bind to FRα-coated beads in most likely a non-specific manner. Together, these suggest that background non-specific binding of beads to cells may be a limitation of this methodology. We employed two approaches to evaluate whether antigen-coupled beads could detect antigen-reactive antibody-expressing cells in PBMC samples.

Our first approach was to “spike” human PBMC with Sp2/0-MOv18 B cells at different frequencies. We demonstrated that FRα^+^ fluorescent beads could identify antibody-expressing cells among PBMC populations when these antigen-reactive cells were present at higher frequencies in human blood. We also found that different bead types had different specific recognition and background recognition profiles. The proportion of actual positive B cells among all bead-bound cells decreased with lower frequencies of FRα+/Sp2/0+ B cells among PBMCs. When B cells were found in high frequencies (≥1:100) in human blood, the proportion of Actual positive events, the specific B cells recognized by the beads, ranged from 5 to 75% of the total bound cells, with the SA-Blue 1.1 µm LC-LC biotin beads able to detect the highest proportion (75%). Although fluorescent activated cell sorting could enable separating a single cell from a heterogeneous population, the detection of cell populations with a low frequency remains challenging and presents a technical limitation with different protocols (33, 34). Consistent with previous studies, here, we observed great variability in the acquired numbers of cells when analyzing samples with very low antigen-specific B cell frequencies. This implies that only active and high frequency humoral responses may be readily studied in the present context, while the detection of low frequency B cells represents an inherent limitation of this methodology and requires further optimization. As a next step, we asked whether using this protocol, fluorescent beads coated with melanoma cell line antigens may be able to identify antigen-reactive B cells in individuals suffering of malignant melanoma. Using melanoma cell line surface antigen-coated fluorescent beads, we expressed a monoclonal antibody that bound to melanoma cell line protein-coated beads. This suggested that antigen-reactive B cells in human blood may be singled out using fluorescent beads.

The selection of single antigen-specific B cells and the identification of their expressed antibodies is critical to gaining a deeper understanding of the nature and functions of active human humoral immune responses. Identification of B cells, as well as cloning and production of their heavy and light chain matched antigen-specific monoclonal antibodies thus remain highly desirable in immunology research and antibody discovery. Consequently, discovery of antigen-specific B cell clones forms the focus of numerous high- and low-throughput approaches (12–14). Our bead-based protocol may provide an alternative, readily applicable means for exploring human B cells by facilitating the study of single B cell-derived antibodies and their functional profiles. Since the frequency of antigen-specific B cells in the human circulation remains ≤1% of the total B cell populations (35), increasing the probability for more specific selection of such low-frequency B cells remains a major challenge with this and many other available technologies. Future efforts may help improve specific selection by incorporating additional selection markers such as beads of multiple fluorophores (31), or B cell activation markers to single out BCR-activated cells more likely to be matured antibody-expressing clones (36, 37). An alternative strategy may entail an additional imaging tool to verify selection of single antigen-reactive B cells subsequent to cell sorting (38). Furthermore, adjusting the cloning process to identify antibodies of different subclasses or specificities or from specific B cell subsets could improve the chance of detecting clones and clonal families in certain diseases, in which immunological conditions may promote specific antibody profiles (39–42). Individually or combined, such approaches may help increase the chances of clonal selection.

In summary, we describe the establishment of a methodology to identify single antibody-expressing cells and to produce and test their sequenced recombinant antibodies in a workflow that may be readily applicable in any basic and translational immunology laboratory setting. This single cell-to-functional antibody strategy may open the way for new opportunities to analyze B cells and their antibody profiles at the single cell level and may be potentially applied to help unravel diverse humoral immune repertoires in blood and tissues and in different health and disease conditions.

## Ethics Statement

Human immune cells were isolated from the venous blood of human volunteers. Specimens were collected with informed written consent in accordance with the Declaration of Helsinki.

## Author Contributions

SK, PK, KL, and FN conceived the study, and IC, KI, SC, PK, KL, FN, AT, and SK designed the methodology. IC, KI, PK, SC, SL, MF, and AC acquired data, generated materials, or helped with the data analysis and interpretation. SK, PK, KI, IC, and SC wrote the manuscript. IC, KI, SC, SL, MF, AC, JS, AT, FN, PK, KL, and SK discussed and interpreted the data and edited the manuscript. SK supervised the study, led and coordinated the project.

## Conflict of Interest Statement

SK and JS are founders and shareholders of IGEM Therapeutics Ltd. FN is an employee of Sanofi US. All other authors declare no conflicts of interest.
