# Supplementary material for: Evaluation of Antigen-Conjugated Fluorescent Beads to Identify Antigen-Specific B Cells
Source: Front Immunol. 2018 Mar 23;9:493. doi: 10.3389/fimmu.2018.00493 (PMC5876289; doi:10.3389/fimmu.2018.00493)
Supplement: Supplementary file 1 [file table_1.docx]

**Supplementary Table 1.** Characteristics of Fluorescent Beads

| **Microsphere Name** | **XMAP LumAvidin Microspheres**  (LumAvidin 5.6 µm) | **Sphero Streptavidin-Coated Fluorescent particles, Nile Red**  (SA-Red 0.5 µm) | **Sphero Avidin-Coated Fluorescent particles, Nile Red** (A-Red 0.8 µm) | **Sphero Streptavidin Coated Fluorescent particles, Blue**  (SA-Blue 1.1 µm) |
| --- | --- | --- | --- | --- |
| **Manufacturer** | Luminex | Spherotec | Spherotec | Spherotec |
| **Catalogue number** | Cat No L100-150-01 | Cat No SVFP-0556-5 | Cat No VFP-0856-5 | Cat No SVFP-1068-5 |
| **Bead size** (size range) | 5.6 µm | 0.5 µm (range 0.4-0.6 µm) | 0.8 µm (range 0.7-0.9 µm) | 1.1 µm (range 1.0-1.9 µm) |
| **Coating** | Avidin | Streptavidin | Avidin | Streptavidin |
| **Fluorochrome** (detection channel) | APC (APC) | Nile Red (PE) | Nile Red (PE) | Blue (APC) |
| **Beads/ml** | >2.5x10^6^ | 1.45x10^10^ | 3.5x10^9^ | 1.8x10^9^ |
| **Biotin binding sites/bead** | >6.7x10^6^ | 3x10^4^ | 5x10^4^ | 2x10^5^ |
| **Beads per test** (total µl per test) | 5x10^4^ (20 µl) | 3x10^7^ (2 µl) | 17.5x10^6^ (5 µl), 7x10^6^ (2 µl) | 3.8x10^6^ (2 µl) |
| **Biotin binding sites/test in** µl | 3x10^11^/20 µl | 9x10^11^/2 µl | 8x10^11^/5 µl or 3.5x10^11^/2 µl | 7.6x10^11^/2 µl |
