# Supplementary material for: Evaluation of Antigen-Conjugated Fluorescent Beads to Identify Antigen-Specific B Cells
Source: Front Immunol. 2018 Mar 23;9:493. doi: 10.3389/fimmu.2018.00493 (PMC5876289; doi:10.3389/fimmu.2018.00493)
Supplement: Supplementary file 2 [file table_2.docx]

**Supplementary Table 2.** Background binding of fluorescent beads to CD19^+^ B cells from human PBMC after incubation of the beads in the presence of the indicated agents or cell treatments

| **Blocking Agent** | **A-Red 0.8 µm microspheres** | **SA-Blue 1.1 µm microspheres** |
| --- | --- | --- |
| BSA 1% | 8.3% | 0.3% |
| FCS 10% | 5.18% | 0.34% |
| Goat serum 10% | 0.7% | 0.34% |
| BlockAid | 1.07% | 0.72% |
| Milk 5% | 1.12% | 0.76% |
| Carbon-free blocking | 7.46% | 1.66% |
| Biotin | 7.0% | 1.48% |
| Avidin-treated B cells + 1% BSA | 5.7% | 1.32% |
| Avidin-treated B cells + 10% FCS | 2.7% | 0.71% |
| Avidin-treated B cells + 10% Goat serum | 1.2% | 1.77% |
| Avidin-treated B cells + BlockAid | 2.0% | 0.76% |
