# Supplementary material for: Evaluation of Antigen-Conjugated Fluorescent Beads to Identify Antigen-Specific B Cells
Source: Front Immunol. 2018 Mar 23;9:493. doi: 10.3389/fimmu.2018.00493 (PMC5876289; doi:10.3389/fimmu.2018.00493)
Supplement: Supplementary file 3 [file table_3.docx]

**Supplementary Table 3.** Detection efficiency of antibody-expressing B cells in PBMC samples using different fluorescent bead types of different sizes

| **Beads** | **Frequency of Sp2/0-MOv18**  **B cells** | **Actual positive events %**  **[TP/(TP+FP)]x100** | **Actual true events**  **%**  **[TP/(TP+FN)]x100** |
| --- | --- | --- | --- |
| A-Red 0.8 µm  LC-LC biotin, 1 µl | 1:34 | 49 | 9.4 |
|  | 1:651 | 7.5 | 18 |
|  | 1:4683 | 0.7 | 18 |
|  | 1:8422 | 0.5 | 25 |
| SA-Blue 1.1 µm  LC-LC biotin, 1 µl | 1:43 | 75 | 15 |
|  | 1:304 | 34 | 21 |
|  | 1:2325 | 4 | 21 |
|  | 1:50000 | 0 | 0 |
| LumAvidin 5.6 µm  LC-LC biotin, 50 µl | 1:54 | 35 | 5 |
|  | 1:408 | 6 | 9 |
|  | 1:3846 | 0.3 | 8 |
|  | 1:20000 | 0 | 0 |
| A-Red 0.8 µm  PEG12 biotin, 5 µl | 1:103 | 6.4 | 43 |
|  | 1:360 | 2.2 | 60 |
|  | 1:2137 | 0.6 | 77 |
|  | 1:8333 | 0.07 | 33 |
| A-Red 0.8 µm  PEG12 biotin, 2 µl | 1:125 | 5.2 | 17 |
|  | 1:431 | 2.6 | 42 |
|  | 1:3703 | 0.3 | 40 |
|  | 1:12500 | 0.01 | 37 |
| SA-Blue 1.1 µm  PEG12 biotin, 5 µl | 1:60 | 28.7 | 40 |
|  | 1:363 | 4.9 | 34 |
|  | 1:2394 | 0.5 | 57 |
|  | 1:10000 | 0.02 | 100 |
| LumAvidin 5.6 µm PEG12 biotin, 30 µl | 1:48 | 48 | 8 |
|  | 1:307 | 19 | 13 |
|  | 1:2738 | 3 | 15 |
|  | 1:33000 | 1.25 | 33 |

TP: true positive; FN: false negative; FP: false positive
