# Supplementary material for: Evaluation of Antigen-Conjugated Fluorescent Beads to Identify Antigen-Specific B Cells
Source: Front Immunol. 2018 Mar 23;9:493. doi: 10.3389/fimmu.2018.00493 (PMC5876289; doi:10.3389/fimmu.2018.00493)
Supplement: Supplementary file 4 [file data_sheet_1.docx]

**
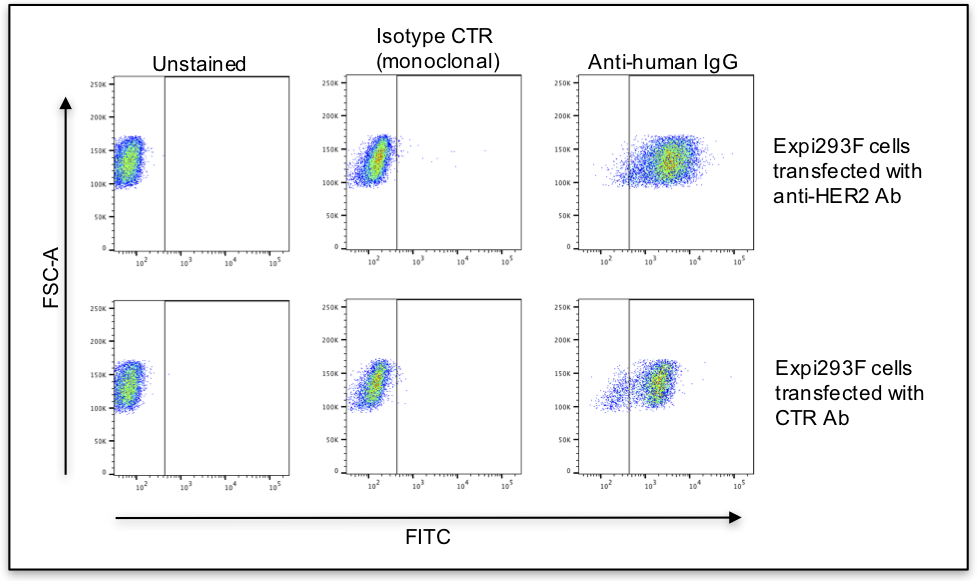
Supplementary Figure 1:** Flow cytometric analyses of Expi293F cells transfected with anti-HER2 antibody (top diagrams) or control antibody (anti-chondroitin sulfate proteoglycan 4, CSPG4) were stained with isotype control antibody or goat anti-human IgG FITC antibody. The gate introduced in each dot plot indicates positive antibody staining above controls.


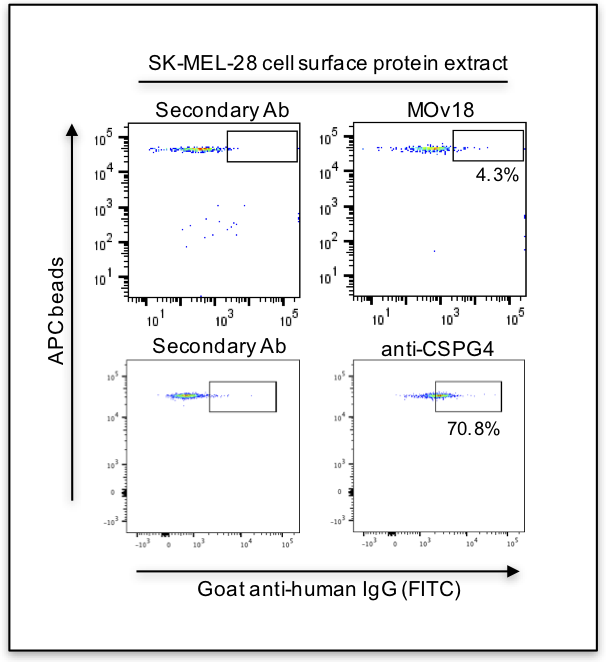


**Supplementary Figure 2:** Biotin-conjugated proteins extracted from the human melanoma cell line SK-MEL-28, coated on fluorescent microspheres (APC) were incubated with secondary antibody only control, MOv18 or anti-CSPG4 antibodies. The percentage of antibody binding beads are indicated in each dot plot.
